# Shift Work Predicts Increases in Lipopolysaccharide-Binding Protein, Interleukin-10, and Leukocyte Counts in a Cross-Sectional Study of Healthy Volunteers Carrying Low-Grade Systemic Inflammation

**DOI:** 10.3390/ijerph182413158

**Published:** 2021-12-14

**Authors:** Aisha Q. Atwater, Lilly Cheng Immergluck, Alec J. Davidson, Oscar Castanon-Cervantes

**Affiliations:** 1 Department of Neurobiology and Neuroscience Institute, Morehouse School of Medicine, Atlanta, GA 30310, USA; atwater_aisha@yahoo.com (A.Q.A.); adavidson@msm.edu (A.J.D.); 2 Department of Microbiology, Biochemistry & Immunology, Morehouse School of Medicine, Atlanta, GA 30310, USA; limmergluck@msm.edu; 3 Pediatric Clinical & Translational Research Unit, Morehouse School of Medicine, Atlanta, GA 30310, USA

**Keywords:** shift work, low-grade systemic inflammation, leukocyte proliferation, lipopolysaccharide-binding protein, systemic endotoxemia

## Abstract

The disruption of inflammatory responses is a potential mechanism behind the harmful effects of shift work and is associated with increased risk of hypertension, stroke, obesity, diabetes, and cancer. These responses are linked to the proliferation of leukocytes in shift workers, suggesting a systemic signal as a potential mediator. The purpose of this study was to assess the relationship between systemic inflammation, leukocyte counts, and systemic endotoxemia in samples from a diverse cohort of day workers and shift workers. Participants (normothermic and normotensive) were healthy volunteers, non-smoking, and drug- and medication-free. The following outcomes were measured: C-reactive protein, TNF-α, IL-6, IL-1β, IL-10, leukocyte counts (monocytes, lymphocytes, and neutrophils), and lipopolysaccharide-binding protein (LBP). Risk factors that increase systemic inflammation, such as blood pressure, sleep loss, and cortisol, were also assessed. The results indicated that shift workers slept significantly less than day workers and had significantly increased concentrations of all of the cytokines measured as well as plasma cortisol. Regression models found that after controlling for covariates, shift-work exposure predicted the significant increase observed in IL-10, leukocyte counts, and LBP. Our results suggest that acute increases in low-grade systemic endotoxemia are unresolved during chronic shift-work exposure. This ongoing immune challenge may underlie the disrupted inflammatory responses characteristic of shift-work-related pathologies. Systemic endotoxemia may represent a novel target to investigate the early effects of exposure to shift-work schedules.

## 1. Introduction

In the U.S., it is estimated that nearly 30% of the workforce is exposed to shift-work schedules [1]. Shift work is broadly defined as any work that is scheduled outside of the traditional 9 a.m.–5 p.m. working day. It includes static night shifts, rotating shifts, extended shifts, flex shifts, and frequent international travel across multiple time zones. It is essential to the economy of the world’s never-sleeping society and provides the resources that are needed to meet the growing demand for goods and services.

This labor practice leads to chronic exposure to hazardous work conditions. Chronic exposure to light at night, sleep loss, circadian disruption, and the associated stress that is inherent to the disruptive environment of shift-work schedules weigh heavily on the health of shift workers. These workers are known to experience increased severity or frequency of several pathologies, including multiple cancers [2,3,4,5,6,7], gastric ulcers [8,9], obesity [10], diabetes [11,12], stroke [11], coronary heart disease, atherosclerosis, heart attack [11,13,14,15], autoimmune disease [16,17], and respiratory infections [18,19,20]. The scientific data are strong enough to classify night shift work as likely being carcinogenic to humans [21].

The disruption of inflammatory responses is a potential mechanism behind the harmful effects of shift-work exposure. Inflammation (the body’s initial response to harmful stimuli) is the first step in the healing process, and therefore, it represents a defense mechanism that is essential to health. Uncontrolled inflammation is a common factor among shift-work-related diseases, given the link between aberrant inflammatory responses and cardiovascular disease [22], cancer [23], and metabolic disorders [24].

Despite this evidence, the mechanisms behind disrupted inflammatory control in shift workers are scarcely understood. Studies investigating shift-work exposure-effects typically characterize changes in inflammatory markers (i.e., cytokines release) but do not address the cause(s) of inflammation. The potential sources of inflammation are diverse. Researchers look for small but significant changes that are predictive of shift work-related disease risk in healthy individuals. However, multiple shift work schedules across industries, the complexity of exposure history within and among individuals, and the diversity of study populations pose a challenge to this effort.

One of the small but significant and consistent changes that is reported in shift workers is an observed increase in the number of circulating leukocytes [20,25,26,27], suggesting an ongoing response to a homeostatic challenge. Since distinct leukocyte populations (i.e., monocytes, lymphocytes, and neutrophils) play a paramount role in immune surveillance, cytokine secretion, and inflammatory responses, an increase in leukocytes suggests that systemic signals elicited in response to a potential antigen may be detected early on in shift workers.

One overlooked mechanism that might be involved in shift-work-related leukocyte activation and proliferation is systemic endotoxemia. Systemic endotoxemia is a hallmark of diseases with high incidence or prevalence among shift workers, such as metabolic syndrome, obesity, cardiovascular disease, and diabetes [28,29]. It results from increased intestinal permeability, which may cause bacterial endotoxin (lipopolysaccharide) translocation into the bloodstream. Lipopolysaccharides (LPS) first bind to their molecular transporter, the LPS-binding protein (LBP), which mediates LPS recognition through the CD14:TLR4 pathway [30]. However, the assessment of LPS in serum or plasma has significant challenges [31,32], including its short half-life, and to overcome these limitations, the determination of LBP (a proxy for LPS) is considered to be a better biomarker for endotoxemia [33].

To the best of our knowledge, changes in systemic LBP levels in healthy shift workers have not been reported. Since inflammation is a defense response, the assessment of endotoxemia as a potential systemic signal that may provide the initial stimulus that triggers the proliferation of leukocytes and increased systemic inflammation is necessary.

To address this question, we investigated the relationship between shift-work exposure and the lipopolysaccharide-binding protein (LBP) in a population of healthy young to middle-aged shift workers and day workers. We first assessed risk factors, including blood pressure changes, cortisol levels, and actigraphy-captured sleep loss. We then determined the association between shift-work exposure and levels of circulating cytokines, C-reactive protein (CRP), primary LPS-responsive leukocytes (monocytes, lymphocytes, and neutrophils), and LBP (a proxy for LPS) as an indicator of low-grade systemic endotoxemia.

## 2. Materials and Methods

### 2.1. Study Cohort and Design

This study enrolled 104 participants representing diverse racial/ethnic backgrounds, aged 21–50 years, with no underlying pathology, non-smokers, and who were free of prescription drugs (except contraceptives) from the general population of the Atlanta metropolitan area in the United States. Participants were contacted by phone and by using informational flyers in break rooms, e-mail announcements, internet-based platforms, and during informational sessions in visits to places of work by the study staff. Interested subjects were sent an intake survey/questionnaire that allowed the study staff to determine eligibility based on the answers that were received. The survey asked participants to describe their past and recent shift work exposure (i.e., type of schedule(s), how many night shifts are scheduled per week and for how many years?). Participants were considered healthy if they did not report current or previous medical history of the following: major mood disorders (depression, bipolar disorder, seasonal affective disorder), inflammation-related disorders such as hypertension, arthritis, colitis, etcetera. Participants were not considered healthy, and therefore were excluded, if they had a BMI ≥ 30 (obese), if they reported current symptoms or active treatment for drug dependency, or if they were taking any medication that may interfere with inflammatory responses such as asthma medications, statins, anticoagulants, immunosuppressive drugs, antidepressants, high blood pressure medications, beta-blockers, sleep medications, pain killers, hormonal medications (menopause or hormone replacement therapy), melatonin supplements, and diabetes medications. To minimize bias, intake surveys were sent to the participants before knowledge of exposure history. The study protocol was approved by the institutional review board of the Morehouse School of Medicine, and all of the participants provided written informed consent.

### 2.2. Exposure Criteria

We used rigorous criteria for participant selection and clearly defined exposure prior to the study. This allowed us to minimize selection bias and to avoid confounding results. Such criteria included defining shift work as those schedules that were different from a typical daytime 8–12 h work shift. These schedules included evening shifts, night shifts, or shifts that changed regularly (rotational) but that did not exceed 12 h per shift. This study included shift workers who worked schedules with significant exposure to light at night (at least 6 h worked between 7 p.m. and 7 a.m.) and at least five night shifts per month for no less than one year immediately before the onset of the study. Day workers had no exposure to shift work schedules in the preceding 3-year period, no more than six months of shift work in a lifetime, and no trans-meridian travel (across more than one time-zone) in the three months prior to the study.

### 2.3. Biological Samples, Risk Factors, and Biomarker Assessments

To assess exposure to factors that are known to increase the risk of systemic inflammation, we measured sleep amount, blood pressure, and plasma cortisol as a marker of psychosocial stress. During the enrollment session, the participants were fitted with an actigraphy device (wGT3X-BT, Actigraph LLC, Pensacola, FL, USA). Participants were asked to wear it continuously. The actigraphy tracker collected data on the amount of sleep that the participants had for at least 7 days prior to blood donation. The total sleep time in each 24 h period per participant was recorded continuously and was scored using the Cole–Kripke sleep algorithm by a person who was blinded to the participant’s exposure condition. This algorithm is typically used to score adult populations [34]. The total sleep time (daily average) was reported in minutes. Blood pressure measurements (mm Hg) were taken prior to blood donations, and plasma cortisol was assessed by a Luminex^®^ bead-based assay (see below).

To assess markers of systemic inflammation, leukocyte cell counts, and LBP, the participants were asked to provide two blood samples (~12 h apart) to control for time-of-day effects on the parameters that can show diurnal differences, one before and one after a daily shift. Blood samples were collected using standard phlebotomy procedures (venipuncture of forearm) in EDTA coated tubes. At each collection time, the blood (10 mL) was drawn into sterile Vacutainer^®^ tubes. A subset of the sample was centrifuged at 1500× *g* for 10 min in the processing lab, and plasma was collected and frozen for later analysis and quantification of protein expression by Luminex^®^ bead-based assays.

Commercially available kits from Millipore Sigma (Milli-Plex, Burlington, MA, USA) were used to quantify the proteins that were present in every plasma sample that was collected in the study. The Luminex^®^ assay was conducted according to the manufacturer’s instructions. Using this approach, we quantified the concentration of the following biomarkers: Cortisol, CRP, TNF-α, IL-6, IL-1β, and IL-10. Both, the Cole–Kripke sleep algorithm and the Luminex^®^ bead-based assay are objective, validated methods that strengthen the study design.

A portion of each blood sample that was not centrifuged was immediately shipped to the clinical diagnostic lab to assess three LPS-responsive leukocyte types (monocytes, lymphocytes, and neutrophils), which was carried out using an automated cell counter. Finally, to investigate if evidence of endotoxin could be detected in the systemic circulation of each participant, a subset of the plasma was probed for the lipopolysaccharide-binding protein (LBP) using a solid-phase sandwich ELISA (R&D Systems, Minneapolis, MN, USA) according to the manufacturer’s instructions.

### 2.4. Data Analysis

Initially, we used descriptive statistics to characterize the differences between the day workers and shift workers. Outcomes are reported as the daily mean per group ± standard error (SE). To calculate this mean, the analytes in each blood sample (one taken before and one taken after a daily shift) and blood pressure readings were assessed separately; values were then averaged to produce the diurnal mean per outcome per participant. The Kolmogorov–Smirnov test assessed the normality of distribution in the data. In those outcomes with normal distribution, two-tailed *t*-tests for independent samples or the Chi-square test assessed the differences among day workers and shift workers; if the distribution was not normal, we assessed differences in the means using the Mann–Whitney U test.

Next, multivariate linear regression analysis was used to assess the contribution of shift-work exposure and the associated risk factors as predictors of distinctive markers of systemic inflammation, leukocyte counts, and LBP. The linear mixed models that were tested accounted for the contribution of the following covariates: age, shift-work exposure, hours worked per shift, cortisol concentration, and total sleep time, to the measured effect. All of the analyses, including parameter estimates, test statistics, and *p*-values, were performed using SAS^®^ 9.4 (SAS Institute Inc., Cary, NC, USA).

## 3. Results

### 3.1. Characteristics of the Study Population

**Figure 1:**
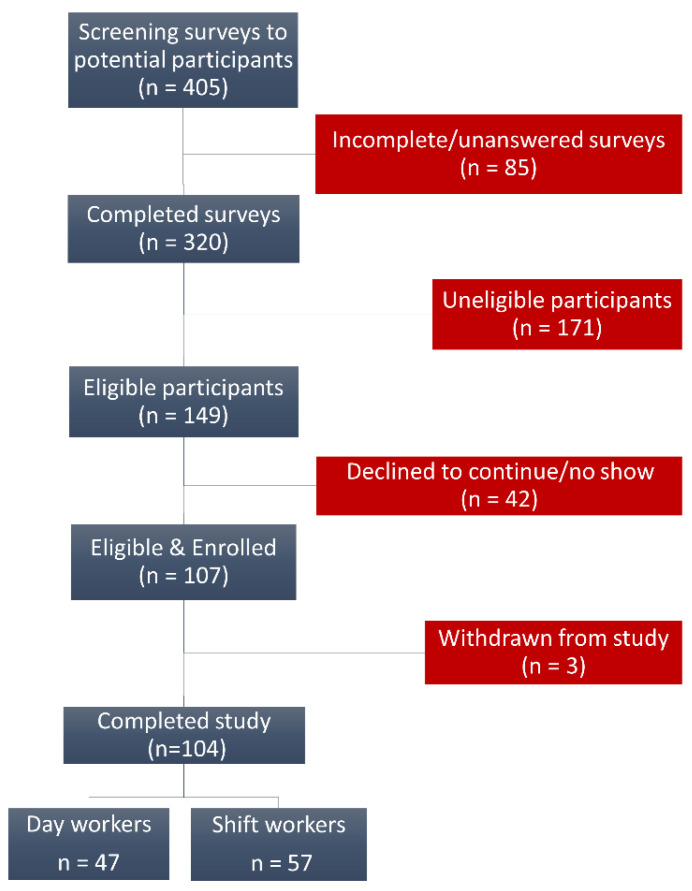
Flow diagram of the recruitment process.

Our recruitment process (Figure 1) allowed us to complete analyses for 104 participants (47-day workers and 57 shift workers). Self-reported racial-ethnic frequencies show a diverse population in which minorities are well represented. Overall, we recruited similar proportions of African Americans (45%) and Whites (42%). Asians (7%), Hispanics (3%), and participants of mixed ethnic background (3%) were also represented. Overall, a large percentage of participants were healthcare workers (84%) and females (89%). We found that shift workers were significantly younger (mean ± SE = 28.26 ± 0.62 years) than day workers (35.32 ± 1.24 years), and we found no statistically significant difference in BMI between the groups.

Shift workers worked a variety of night schedules that were broadly characterized based on the number of years working night shifts and the number of hours worked per shift. Shift workers had an average exposure to night-shift schedules of 3.97 ± 0.51 years, and in addition, they worked significantly longer hours per shift (mean ± SE = 11.78 ± 0.12 h worked per shift) than day workers did (9.36 ± 0.27 h per shift). Table 1 summarizes the compared characteristics between the day workers and the shift workers and a breakdown of the race/ethnic frequencies by exposure group.

### 3.2. Assessment of Blood Pressure, Sleep Amount, and Plasma Cortisol

In addition to shift work as a risk factor, we also evaluated the exposure of the cohort to changes in three factors that are known to increase the risk of systemic inflammation: blood pressure, sleep amount, and plasma cortisol level. In this regard, we found that the blood pressure (both systolic and diastolic pressure) level between day workers and shift workers was not significantly different. However, the shift workers did show a significant decrease in total sleep amount. The mean sleep time for the shift workers was 326.85 ± 8.94 min per day, nearly 70 min less than the sleep time that was scored in the day workers (395.9 ± 13.90). Similarly, plasma cortisol, a stress marker, was significantly increased (39.2%) in the individuals who were exposed to shift work (mean ± SE = 183.07 ± 14.56 ng/mL) compared to the day workers (131.55 ± 10.93 ng/mL). Table 2 summarizes the compared differences in exposure to risk factors between the day workers and the shift workers.

### 3.3. Markers of Systemic Inflammation in Shift Workers

**Figure 2:**
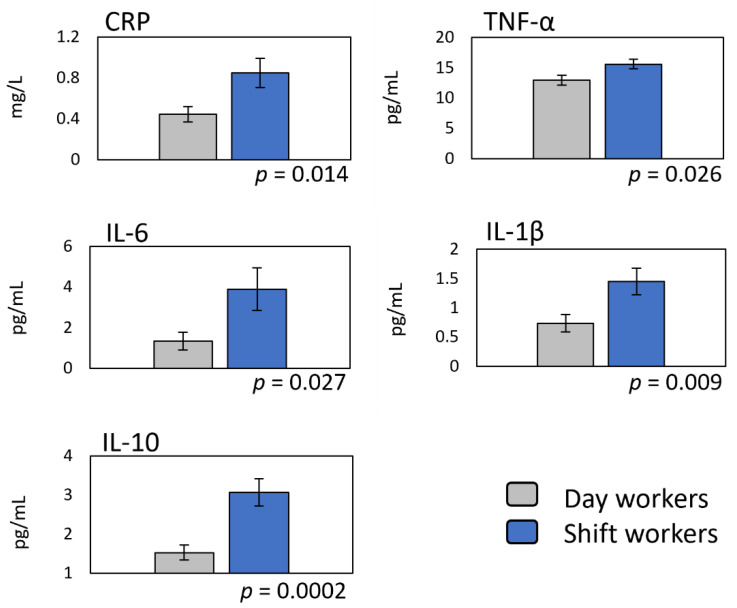
Changes in distinct markers of systemic inflammation as a function of shift-work exposure. Data (*n* = 104; 57 shift workers) represent group means ± standard error. Differences were assessed by the student’s *t*-test for independent samples. Respective *p* values indicate statistical significance.

To monitor the degree of the potential responses of the body to the challenges that are imposed by exposure to significant risk factors, we assessed the levels of known markers of systemic inflammation (Figure 2). These included CRP and four major cytokines (TNF-α, IL-6, IL-1β, and IL-10). Compared to the day workers, the shift workers had a CRP concentration that was 93% higher (0.85 ± 0.14 vs. 0.44 ± 0.07 mg/L). Similarly, the shift workers had significantly increased concentrations of TNF-α, which was 20% higher (15.61 ± 0.81 vs. 12.97 ± 0.84 pg/mL), IL-6, which was 190% higher (3.89 ± 1.05 vs. 1.34 ± 0.44 pg/mL), IL-1β, which was 96% higher (1.45 ± 0.23 vs. 0.74 ± 0.15 pg/mL), and IL-10, which was 100% higher (3.07 ± 0.35 vs. 1.53 ± 0.19 pg/mL).

**Figure 3:**
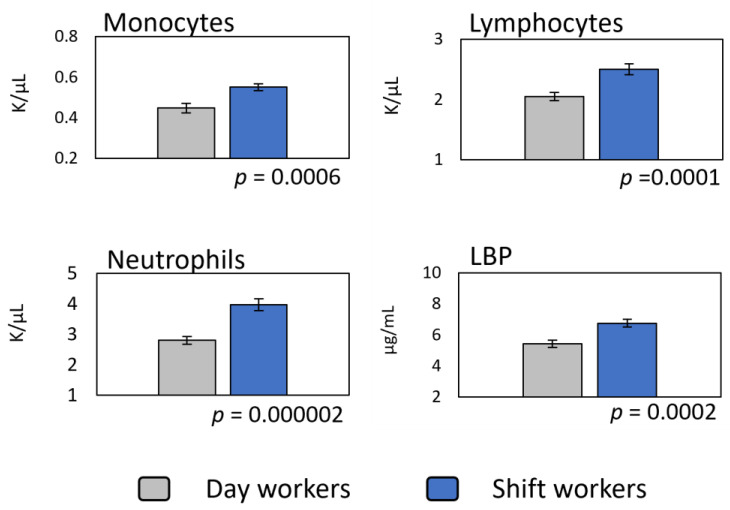
Assessment of leukocyte proliferation and systemic endotoxemia as a function of shift-work exposure. Data (*n* = 104; 57 shift workers) represent group means ± standard error. Differences were assessed by the student’s *t*-test for independent samples. Respective *p* values indicate statistical significance.

These observed increases in cytokines in the shift workers are consistent with a proliferation of leukocytes in the bloodstream (Figure 3). Although the proteins that were found to be elevated in shift workers are secreted by many cells in the body, three immune cell types: monocytes, lymphocytes, and neutrophils, account for most of these cytokines. Thus, when compared to the day workers, the shift workers had mean counts of monocytes that were higher by 23% (0.551 ± 0.02 vs. 0.448 ± 0.02 K/μL), lymphocytes, which were higher by 22% (2.504 ± 0.09 vs. 2.053 ± 0.07 K/μL), and neutrophils, which were higher by 41.5% (3.965 ± 0.19 vs. 2.802 ± 0.13 K/μL).

Because monocytes, lymphocytes, and neutrophils all respond to LPS and because these cells are significant contributors to the observed systemic cytokine increase in shift workers, we assessed changes in systemic endotoxemia as a function of shift-work exposure. We assessed LBP (a proxy for lipopolysaccharide) in the plasma samples (Figure 3). When compared to the day workers, the shift workers had a mean concentration of LBP that was 26% higher (6.82 ± 0.27 vs. 5.42 ± 0.24 μg/mL).

### 3.4. Relationship between Shift-Work Exposure, Leukocyte Counts, and Systemic Endotoxemia

Finally, we tested multivariate linear mixed models to assess the combined effect of shift work together with the risk factors that contribute to changes in systemic inflammation, leukocyte counts, and LBP concentration. When covariates were included in the models to account for their effect, we found overall regression models that were statistically significant for the following outcomes: IL-6, IL-10, monocytes, lymphocytes, neutrophils, and LBP. In all of these models, except for IL-6, shift-work exposure was a significant predictor of the outcome. IL-6 was instead significantly predicted by the level of plasma cortisol (*p* = 0.004). In addition to the overall model fit parameters, Table 3 summarizes the shift-work exposure effect estimates for all of the outcome measures, including the 95% confidence interval (CI) and the estimated *p*-value of shift-work exposure as a predictor.

## 4. Discussion

### 4.1. Low-Grade Systemic Inflammation in Shift Workers Is Promoted by Multiple Factors and Does Not Require a Lifetime of Exposure to Emerge

The inflammatory effects that are reported here may underlie the known increase in the incidence and prevalence of many pathologies such as diabetes, stroke, cardiovascular disease, and several cancers in shift workers [2,3,4,5,6,7,8,9,10,11,12,13,14,15,16,17]. However, these inflammatory effects can also be considered symptoms of an existing imbalance in homeostatic regulation. Thus, rather than the root cause of the health maladies impacting shift workers, inflammation can be initially seen as a defense response to the acute homeostatic insult that is posed by the challenges that are inherent to shift-work exposure. Even though shift work can exert its effects over long periods of time, acute changes can be systematically assessed. Thus, a lifetime of exposure is not needed to reveal adverse health consequences.

In this study, the average exposure to shift work schedules was 3.97 years (median = 3, range = 1–20). This exposure level is sufficient to demonstrate a pervasive increase in most of the inflammatory outcomes that were measured. Behavioral factors that are inherent to shift work (i.e., sleep loss, stress, etcetera) and specific antigens that we all face daily challenge the immune system constantly. Under the conditions of low-grade systemic inflammation, pathological responses that are incapable of resolving an immune challenge can develop. In fact, experimental models of obesity, diabetes, and cardiovascular disease [28,29,35] have shown that an overactive immune system may reveal pathological responses when challenged. In animal models of shift work, we previously demonstrated that disrupted inflammatory responses could be uncovered by an ex vivo endotoxin challenge [36,37,38]. Thus, in shift workers, chronic, low-grade systemic inflammation, such as the inflammation that is reported here, can be characterized as an unresolved immune challenge. The source of this challenge is antigenic, but the behavioral and socio-economic factors that are underlying these changes should also be considered. In the end, an inflammatory event that cannot be resolved would be expected to increase the incidence and prevalence of pathologies in which controlling inflammation is key to the outcome.

Our geographical position in the Metropolitan Atlanta area and our outreach programs as a historically Black institution allowed us to increase the representation of minorities in our research cohort (see Table 1). We aimed to better represent the composition of the population whose health risk and prognosis we seek to understand; thus, we report the frequencies of self-identified ethnic/race groups. It is essential to increase the participation of these groups in research studies. After all, shift work does not impact people’s health equally. Indeed, minorities are more than twice as likely to participate in shift work as Whites [21]. Underlying health conditions in shift workers who experience historic socio-economic disadvantages will likely be aggravated. In the U.S., for example, 14.6% of the population, or about 47.4 million people, are identified as African American, a population that has a higher prevalence and incidence of chronic diseases [39,40]. As previously mentioned, many of these pathologies are characterized by an increase in chronic systemic inflammation. We did not attain the power necessary to include race as a covariate in this study; however, some studies have specifically reported the shift work effects that are associated with race [41,42,43,44,45]. Significantly, race contributes to increased systemic inflammation among racial-ethnic minorities [46,47,48,49]. Thus, if Blacks, Hispanics, Asians, and other minorities are adequately represented in shift-work studies, exacerbated low-grade systemic inflammation and its consequences may be expected to be even worse than what is typically reported. Further research on the association between ethnicity/race and disrupted inflammation in shift workers is warranted.

This study finds significant differences in many of the risk factors brought about by shift-work exposure. It is necessary to characterize these homeostatic responses now if the prediction and prevention of shift work-related disease is the goal, and this is essential given the vast diversity of shift work populations, shift schedules, stress responses, and lifestyles (sleep-adjusting routines; social and economic determinants of health; dietary, alcohol, and caffeine consumption preferences; etcetera.) that drive changes in inflammation and that are nearly impossible to control for in field studies. In this study, we reported that shift workers show consistent increases in cytokine release, sleep loss, and plasma cortisol, all of which are factors that are known to contribute to a state of low-grade systemic inflammation [50,51,52,53,54].

In this regard, subtle differences in blood pressure that are not statistically significant now may still result in a higher risk of future pathology for shift workers (see Table 2). This is not a new idea. For example, C-reactive protein levels (found to be significantly elevated in this study by 93% in shift workers) are known to be significantly and independently associated with the future development of hypertension [55]. At least one laboratory-based study has reported increased systemic levels of the C-reactive protein in an experimental model of short-term circadian misalignment in healthy career shift workers [50]. Our findings are consistent with these observations and indicate that acute changes in systemic inflammation, from leukocyte proliferation to increased cytokine release, do not require decades of exposure to shift work to emerge.

Increased inflammation has been reported in shift workers before [56,57,58,59]. Our findings show significantly increased levels of distinctive cytokines (TNF-α, IL-1β, IL-6, and IL-10) in the systemic circulation in our cohort of shift workers (see Figure 2). The increased release of these and other cytokines are known to be associated with sources of inflammation that are linked to shift-work exposure. For example, TNF-α, and IL-1β are pro-inflammatory cytokines with similar kinetics, while IL-10 is anti-inflammatory and exerts its actions over a more extended timeframe [60,61]. Both TNF-α and IL-1β promote sleep, and their levels increase after sleep deprivation [62]. The increase in IL-10 that is reported here is predicted by shift-work exposure (see Figure 2; Table 3), indicating that solid anti-inflammatory action is needed to inhibit the pro-inflammatory drive of TNF-α, IL-6, and IL-1β. IL-10 is a potent inhibitor of TNF-α, IL-1β, and IL-6 [63]. IL-6 release was also increased in shift workers. When IL-6 is activated through monocytes, it exerts inflammatory activity. On the other hand, IL-6 elicits anti-inflammatory action when it is produced by muscular contraction [64]. In this study, the increase in IL-6 was significantly associated with plasma cortisol, while its association with shift-work exposure was not significant. This observation illustrates the complexity of the intertwined pathways that directly or indirectly impact the regulation of IL-6 through shift work. Stress and sleep loss (risk factors inherent to shift work) are indeed known to increase IL-6 and cortisol release [65,66], contributing to an overall state of low-grade systemic inflammation.

In addition to these regulatory effects, circadian disruption is also inherent to shift-work exposure. Circadian clocks regulate some critical aspects of immune function, including the timing of the secretion of several cytokines, chemokines, and growth factors, including IL-1β, TNF-α, IL-2, GM-CSF, IL-6, MCP-1, IFN-γ, IL-10, and CCR2 [67,68,69,70,71]. In a previous study, we showed that some ex vivo responses to elicited endotoxin-challenge in humans are also regulated by circadian mechanisms [72]. However, this study did not investigate diurnal variations in markers of systemic inflammation, and the two measurements of each outcome that were taken (~12 h apart) provided the best estimate by attempting to control for variations due to diurnal and circadian rhythmicity. Because shift workers were enrolled at different stages of their circadian rhythm, it was impossible to predict the effect of shift-work exposure on the timing of group peak-values of those outcomes known to oscillate diurnally. Others have investigated the role of circadian disruption in the regulation of immune function and shift work-induced inflammation [50,73,74,75,76,77,78,79].

### 4.2. Systemic Endotoxemia as a Potential Mediator of Shift-Work-Related Effects

As we mentioned, complex signaling pathway interactions are involved in regulating cytokine synthesis and release. Thus, although informative of the effect of shift work on the overall inflammatory state, the assessment of inflammation in plasma or serum has limited value unless the upstream pathways that initiate the cascade of cytokine release are investigated. Under specific circumstances and at different stages of activation, the cytokines that were investigated in this study are released by monocytes, lymphocytes, and neutrophils. These cells were also found to be increased significantly as a function of shift-work exposure. These immune system cells are an essential component of the vast surveillance network that is necessary to understand sources of inflammation and the mechanisms that keep it under control.

Given the increase in cytokine release reported here, the proliferation of leukocytes in shift workers was expected. Indeed, we found that the number of these leukocytes in circulation is predicted by shift-work exposure (see Figure 3; Table 3). Others have shown an increase in distinctive leukocyte counts in shift workers [20,25,26,27,57,59,80], but little attention has been given to investigating the potential mechanism behind this proliferation.

Monocytes, lymphocytes, and neutrophils proliferate when they are activated through molecule-specific receptors. TLR-4, the LPS receptor, is mainly expressed in monocytes [81]; however, lymphocytes and neutrophils can also mount LPS-elicited responses [82,83], contributing to cytokine release and overall systemic inflammation. Even though not all cells are activated simultaneously, the cascade of events that is triggered by LPS-elicited responses may result in the proliferation of all of these cells. Indeed, pathologies in which low-grade systemic inflammation is prevalent are characterized by an increase in systemic endotoxemia and proliferation of distinctive leukocytes [28,33,35,84,85,86]. An increase in circulating bacterial endotoxin in shift workers could explain the proliferation of leukocytes and the increased systemic inflammation that follows.

Along with sleep loss and circadian disruption, the psychosocial stress that is posed by exposure to shift work schedules is likely to induce significant homeostatic changes. One possibility of such changes is an increase in intestinal membrane permeability, resulting in elevated bacterial translocation and its products into the systemic circulation. With increased membrane permeability, the body can access more water and energy resources that are needed to cope with the challenges that are posed by a potential stressor [87]. We reasoned that the systemic inflammation and leukocyte proliferation that are elicited by shift work, a significant stressor, may be associated with increased bacterial endotoxin in circulation. The assessment of LBP (a proxy for LPS) in plasma samples reported here found that shift workers have an increase of 26 % in plasma LBP compared to day workers. This increase is predicted by shift-work exposure in our regression models.

We hypothesized that the increased intestinal permeability that is induced by the combined effects of sleep loss, circadian disruption, and stress associated with shift-work exposure may result in LPS translocation into the systemic circulation (detected by an increase in systemic LBP). The circulating LPS would increase leukocyte cell counts, particularly of those cell types that are able to mount LPS-elicited responses.

To the best of our knowledge, this is the first report that links a potential increase in the lipopolysaccharide-binding protein and shift-work exposure. Indeed, significant regression models that were adjusted for covariates confirmed that an increase in LBP levels, IL-10, and leukocyte counts can be predicted by exposure to shift work schedules (see Table 3).

The mechanisms and pathways that determine cytokine release are complex and diverse. At the same time, the level of exposure to risk factors that promote inflammation, such as changes in sleep loss, shift frequency, duration, direction, etcetera, across studies are also complex and diverse. This intrinsic complexity of interactive factors suggests that the common upstream pathways that trigger inflammation, such as low-grade systemic endotoxemia, may provide a novel target for the investigation of the early effects of shift-work exposure.

Further research is required to investigate the association between LBP and leukocyte responses that are specific to LPS-elicited activation in shift workers. A strong association or dependency between systemic endotoxemia and monocytes, lymphocytes, and neutrophils is likely to emerge if the isolation of antigen-specific activated cells is investigated in future studies. Additionally, an investigation of circulating levels of antigen-specific activation markers such as CD14 and CD163, among others, is warranted.

Finally, the effect of acute and chronic exposure to circulating endotoxin in shift workers also deserves further research. Bacterial translocation is not exclusive to shift-work exposure, but the effects of low-grade systemic endotoxemia are well known. Chronic exposure to circulating endotoxin in otherwise healthy shift workers may either exacerbate or suppress inflammatory responses. The characterization of these responses after exposure to shift work can be tested experimentally both in animal models and in humans through established ex vivo endotoxin response assays [36,37,38,72]. Monocytes, for example, are significant contributors to the release of TNF-α, IL-1β, and IL-6. They may also be rendered tolerant to LPS stimuli [88], modifying cytokine release. Interestingly, disrupted LPS-elicited responses are known to explain both tolerance and priming, leading to uncontrolled inflammation [85,89,90,91]. With increased shift-work exposure, these effects become chronic, suggesting that systemic endotoxemia may become a critical factor defining if, how, and when inflammatory control is disrupted, eventually leading to an increased incidence and prevalence of disease.

### 4.3. Strengths and Limitations

The strengths of this study include the cross-sectional design, minimization of bias through the rigorous definition of exposure criteria, and the use of objective and validated methods and technologies. A significant strength lies in the racial/ethnic diversity of our cohort, which included a large proportion of minorities, who are typically underrepresented in clinical studies. The limitations of the study include lacking control of some confounding factors such as diet consumption and lighting conditions that may impact inflammatory outcomes. We struggled to enroll healthy shift workers with more prolonged exposure to shift-work schedules (10 or more years of exposure). This fact limits the ability to assess the role of exposure duration on inflammatory outcomes, a question that is paramount to shift work organization. Investigation of the effects of real-life shift work on inflammatory responses should also be expanded to populations outside of the healthcare sector. In this study, healthcare workers were overrepresented. This was likely due to our ability to schedule blood donations directly at different hospitals right before or after working hours. In contrast, non-healthcare workers had to schedule out-of-work time to travel to our donation site, and many times, this resulted in an inconvenience that discouraged participation or completion of the study. Shift-work effects may thus differ by industry and may depend on chronic exposure duration, shift length, shift frequency, and shift direction. All of these variables require specific investigation and may limit the applicability of our findings.

## 5. Conclusions

Overall, the results of this study show that in young to middle-aged shift workers who have been deemed to be healthy, exposure to shift-work schedules results in the development of a state of low-grade systemic inflammation, the proliferation of leukocytes, and a significant increase in systemic lipopolysaccharide-binding protein (LBP). These results also indicate that the effects of shift-work exposure are pervasive and can be detected early on as acute homeostatic changes across several physiological systems that are related to immune surveillance, sleep homeostasis, acute phase, and psychosocial stress responses. Notably, the increases in IL-10; monocyte, lymphocyte, neutrophil counts; and LBP concentration are all predicted by exposure to shift-work schedules. This observation provides a potential link between a specific systemic signal that is capable of immune activation in healthy shift workers and disrupted inflammatory responses for the first time.

Although challenging, characterizing these changes in healthy shift workers who are carrying systemic inflammation is relevant. This effort should lead to the early identification of biomarkers for the risks that are associated with these labor practices with the potential to predict and prevent increased hazards to health.

## Figures and Tables

**Table 1:** Characteristics of the study population.

	Day Workers *n* = 47	Shift Workers *n* = 57
Asian (%)	6.4	7
Black (%)	57.4	35
Hispanic (%)	2.1	3.5
Mixed (%)	2.1	3.5
White (%)	32	51
Gender (% females)	82	89
Occupation (% healthcare)	77	84
BMI (kg/m^2^)	25 ± 0.38	24.03 ± 0.37
* Age (years)	35.32 ± 1.24	28.26 ± 0.62
* Shift duration (h)	9.36 ± 0.27	11.78 ± 0.12
* Shift-work exposure (years)	0	3.97 ± 0.51

Except as indicated otherwise, values are expressed as means ± standard error. * Significant difference (*p* < 0.05) between shift workers and day workers assessed by independent *t*-test, Mann–Whitney U test, or Chi-square test.

**Table 2:** Factors that increase risk of systemic inflammation.

	Day Workers *n* = 47	Shift Workers *n* = 57
Systolic blood pressure (mm Hg)	120.73 ± 1.79	121.64 ± 1.36
Diastolic blood pressure (mm Hg)	76.21 ± 1.10	78.21 ± 1.09
* Total sleep time (min per day)	395.9 ± 13.90	326.85 ± 8.94
* Plasma cortisol (ng/mL)	131.55 ± 10.93	183.07 ± 14.56

Values are expressed as means ± standard error. * Significant difference (*p* < 0.05) between shift workers and day workers assessed by independent *t*-test.

**Table 3:** Multivariate regression estimates of the effect of shift-work exposure on inflammatory outcomes, leukocyte counts, and LBP as a proxy for systemic endotoxemia after adjusting for covariates.

Outcome Variables	Regression Coefficient	95% (CI)	*p*	Overall Model Fit
CRP (mg/L)	0.415	−0.145–0.844	0.058	*F* (5, 98) = 1.46, *R*^2^ = 0.06, *p* = 0.22
TNF-α (pg/mL)	3.081	0.165–6.001	2.096	*F* (5, 98) = 1.78, *R*^2^ = 0.07, *p* = 0.14
IL-1β (pg/mL)	0.816	0.104–1.528	0.025	*F* (5, 98) = 1.80, *R*^2^ = 0.07, *p* = 0.13
IL-6 (pg/mL)	2.542	−0.379–5.463	0.087	*F* (5, 98) = 3.66, *R*^2^ = 0.13, *p* = 0.008
* IL-10 (pg/mL)	1.674	0.617–2.732	0.002	*F* (5, 98) = 3.56, *R*^2^ = 0.12, *p* = 0.009
* Monocytes (K/μL)	0.117	0.047–0.186	0.001	*F* (5, 98) = 4.19, *R*^2^ = 0.15, *p* = 0.003
* Lymphocytes (K/μL)	0.328	0.038–0.618	0.027	*F* (5, 98) = 4.93, *R*^2^ = 0.17, *p* = 0.001
* Neutrophils (K/μL)	1.121	0.511–1.731	0.000	*F* (5, 98) = 5.65, *R*^2^ = 0.18, *p* = 0.0004
* LBP (μg/mL)	1.312	0.422–2.202	0.000	*F* (5, 98) = 5.43, *R*^2^ = 0.18, *p* = 0.0005

* Outcome significantly predicted by shift-work exposure AND modeled by a significant regression equation. In addition to shift-work exposure, models were adjusted for age, daily shift duration, sleep amount, and cortisol level.

## Data Availability

Data from this study are available from the corresponding author upon reasonable request.
